# Cerebral haemodynamic responses to inspiratory muscle work

**DOI:** 10.1113/EP092840

**Published:** 2025-09-11

**Authors:** Andrew H. Ramsook, Morgan A. Hughes, Wyatt W. Pruter, Kevin L. Webb, Ali Ataie, Chad C. Wiggins, Gabrielle A. Dillon, Sarah E. Baker, Michael J. Joyner

**Affiliations:** ^1^ Department of Anesthesia and Perioperative Medicine Mayo Clinic Rochester Minnesota USA; ^2^ Mayo Clinic Alix School of Medicine, Mayo Clinic Rochester Minnesota USA; ^3^ Radboud University Medical Center, Radboud University Nijmegen The Netherlands; ^4^ Department of Kinesiology Michigan State University East Lansing Michigan USA; ^5^ Department of Health and Kinesiology University of Illinois at Urbana‐Champaign Urbana Illinois USA; ^6^ Department of Physiology & Biomedical Engineering Mayo Clinic Rochester Minnesota USA

**Keywords:** cardiovascular regulation, pressure‐threshold loading, sex difference, work of breathing

## Abstract

Fatiguing inspiratory work has been shown to evoke a sympathetically mediated reflex that has systemic cardiovascular consequences, including increases in heart rate and blood pressure and a decrease in resting limb vascular conductance. Moreover, the response to this reflex appears to be attenuated in females compared with males. It remains to be seen whether this respiratory muscle metaboreflex also exerts an effect on cerebral blood flow. Cerebral blood flow is tightly regulated to maintain homeostasis and critical function. Therefore, it stands to reason that cerebrovascular haemodynamics would not be compromised through this respiratory muscle metaboreflex. We hypothesized that fatiguing inspiratory work would reduce resting limb conductance, but cerebral blood flow would be minimally impaired. Females (34 ± 10 years old, *n *= 12) and males (31 ± 8 years old, *n *= 12) performed a 5 min, high‐intensity bout of inspiratory pressure threshold loading (IPTL) designed to evoke the respiratory muscle metaboreflex. In response to IPTL, mean arterial pressure increased in both males and females (*p <* 0.001), and limb vascular conductance decreased to a greater degree in males than in females (*p =* 0.005). The cerebrovascular conductance index was higher in females (*p =* 0.007) but not affected by IPTL (*p =* 0.417). Our findings suggest that cerebral blood flow is spared from the redistribution of blood flow in response to fatiguing inspiratory work and that this protection is true in both males and females.

## Abstract (highlights)

- **What** **is the central question of this study?**
  The respiratory muscle metaboreflex is thought to ‘steal’ cardiac output from the peripheral muscles to facilitate the demand of the respiratory muscles. Fatiguing inspiratory muscle work might also negatively impact cerebral blood flow.
- **What is the main finding and its importance?**
  Our findings show that activation of the respiratory muscle metaboreflex does not compromise cerebrovascular function. Additionally, the cerebrovascular response to the muscle metaboreflex response is similar between males and females.

## 1 INTRODUCTION

Periods of high‐intensity, fatiguing inspiratory muscle work can elicit a sympathetically mediated pressor reflex, referred to as the respiratory muscle metaboreflex (St Croix et al., 2000). Facilitated by the activation of group III and IV afferent fibres (Haouzi et al., 1999) of the respiratory muscles, this respiratory muscle metaboreflex influences cardiovascular haemodynamics, as evidenced through increases in mean arterial pressure and heart rate (Leahy et al., 2024; St Croix et al., 2000) and a reduction in resting limb vascular conductance (Sheel et al., 2001; Smith, Broxterman et al., 2016). Presumably, this reflex results in a redistribution of blood flow to the fatiguing, active respiratory muscles at the expense of the peripheral limbs. Although the peripheral resting limbs have frequently been the site of study regarding the cardiovascular consequences of the respiratory muscle metaboreflex, effects on cerebral blood flow have received less attention.

Cerebral blood flow is highly regulated to maintain homeostasis. Much attention has been given to understanding the mechanisms that regulate cerebral blood flow at rest, but the addition of whole‐body dynamic exercise provides new challenges to our understanding of cerebral blood flow regulation. In general, cerebral blood flow increases proportionately with progressive exercise intensity until reaching ∼60% maximal oxygen uptake, after which cerebral blood flow will remain constant or even slightly decline (Smith & Ainslie, 2017). The specific alteration of the cerebral blood flow response to high‐intensity exercise (i.e. >60%maximal) appears to be heavily influenced by arterial CO_2_ (Smith, Wildfong et al., 2016), although it is possible that other factors also contribute. One such contributing factor might be the respiratory muscle metaboreflex, because it is thought to redistribute cardiac output to meet the demands of the respiratory muscles (Sheel et al., 2018). It stands to reason that cerebral blood flow would be prioritized and not subject to any ‘theft’ of cardiac output to support the respiratory muscles; however, whether activation of the respiratory muscle metaboreflex via fatiguing diaphragm work exerts an influence on cerebral blood flow remains to be seen.

Additionally, there is existing evidence to suggest that the cardiovascular consequences (e.g. changes in heart rate, mean arterial pressure and limb vascular conductance) of the respiratory muscle metaboreflex are attenuated in females compared with males (Benbaruj et al., 2024; Geary et al., 2019; Leahy et al., 2024; Smith, Broxterman et al., 2016). Combined with findings that posit sex differences in cerebrovascular regulation, including greater cerebral blood flow in females at rest (Robison et al., 2019), there is merit to exploring sex‐based differences in the respiratory muscle metaboreflex responses and cerebral blood flow.

Accordingly, the primary purpose of this study was to explore cerebrovascular haemodynamic responses to high‐intensity, fatiguing inspiratory muscle work. We hypothesized that measures of cerebrovascular haemodynamics would not be compromised in response to the respiratory muscle metaboreflex stimulated through inspiratory pressure threshold loading (IPTL). Furthermore, we considered an additional exploratory aim to gain preliminary insight into potential sex‐based differences in the cerebrovascular hemodynamic responses to the respiratory muscle metaboreflex. We hypothesized that measures of cerebrovascular haemodynamics would be greater in females compared with males, but that inspiratory work via IPTL would have no influence on the responses in either sex.

## 2 MATERIALS AND METHODS

### 2.1 Ethical approval

All study procedures were approved through the Mayo Clinic Institutional Review Board (IRB: 23‐002773) and in accordance with the *Declaration of Helsinki* except for registration in a public database. All participants provided written informed consent before study involvement.

### 2.2 Experimental overview

Study procedures took place over a single visit. Upon arrival at the laboratory, participant height and weight were recorded, followed by spirometry. After instrumentation, participants remained semi‐supine and performed a series of maximal inspiratory pressure manoeuvres from residual volume. Participants were familiarized with the IPTL task until comfortable matching the required pace and intensity. Participants then lay resting, semi‐supine, for a 5‐min baseline period, followed by 5 min of IPTL. Data during baseline and IPTL were divided into 1‐min segments for subsequent analysis.

### 2.3 Participants

Male and female participants with no reported symptoms of cardiovascular, respiratory or musculoskeletal diseases were included in this study. Participants were excluded if the ratio of forced expiratory volume in 1 s (FEV_1_) to forced vital capacity (FVC) was ≤0.70 or if FEV_1_ was ≤80% predicted  . Female participants were tested randomly throughout the menstrual cycle. All female participants were not pregnant, as confirmed via urine pregnancy test on the day of testing.

### 2.4 Pulmonary function

Spirometry was performed according to standard recommendations (Miller et al., 2005) using spirometry modules on a commercially available metabolic cart system (BreezeSuite v.8.5; MGC Diagnostics, St Paul, MN, USA). Participant spirometry data are reported as absolute and percentage predicted values (Bowerman et al., 2023; Quanjer et al., 2012). Inspiratory muscle strength was assessed via maximal inspiratory pressure (MIP) manoeuvres from residual volume (American Thoracic Society/European Respiratory Society, 2002) and also expressed as absolute and percentage of predicted values (Black & Hyatt, 1969). Briefly, MIP manoeuvres were considered valid if they were ≥2 s, meaning that the maximum sustained pressure over 1 s could be recorded.

A minimum of three MIP manoeuvres were performed, and the greatest (i.e. most negative) value was used to determine the target inspiratory load for the IPTL task.

### 2.5 Cardiovascular and respiratory outcomes

Heart rate was recorded continuously via three‐lead ECG (Cardiocap 5, Datex‐Ohmeda, Louisville, CO, USA). Blood pressure was monitored continuously using finger volume‐clamp photoplethysmography (Model 1, Nexfin, BMEYE, Amsterdam, The Netherlands) on the right hand. Manual blood pressure measurements were taken before baseline and IPTL and immediately after IPTL to calibrate and verify photoplethysmography‐derived blood pressure. Inspiratory flow was measured via pneumotachometer (Hans Rudolph 3813, Shawnee, KS, USA) placed on the inspired circuit of the IPTL device. The partial pressure of end‐tidal CO_2_ ($P_{\mathrm{ET},CO_{2}}$) was sampled through a side‐port at the mouth (Cardiocap 5).

### 2.6 Respiratory pressures

Oesophageal pressure (*P*_OES_) and gastric pressure (*P*_GA_) were measured via a dual balloon oesophageal balloon catheter (Guangzhou Yinghui Medical Equipment Co. Ltd, Guangzhou, China). The position of the *P*_OES_ balloon was confirmed via occlusion test (Baydur et al., 1982). Mouth pressure was measured via a port in the inspired circuit. All pressures were connected to calibrated differential pressure transducers (DP15; Validyne Engineering, Canoga Park, CA, USA) connected to a Carrier Demodulator (CD280; Validyne Engineering). Transdiaphragmatic pressure (*P*_DI_) was calculated as the difference between *P*_GA_ and *P*_OES_. Pressure–time products of the mouth (PTP_MO_) and transdiaphragmatic (PTP_DI_) pressures were calculated through the numerical integration of an ensemble‐averaged pressure trace during 30‐s segments of the IPTL trial and multiplied by breathing frequency over the same segment.

### 2.7 Limb blood flow

Femoral artery blood velocity and diameter were measured using duplex ultrasonography via 4 MHz linear‐array Doppler probe (Arietta 70, Hitachi Healthcare; Twinsburg, OH, USA). The outermost envelope of the Doppler signal was transferred into PowerLab via an audio signal conversion (Transcranial Doppler model 500M, Multigon Industries Inc., Elmsford, NY, USA) between the ultrasound unit and PowerLab. Femoral artery blood flow was estimated as the product of mean blood velocity and femoral artery cross‐sectional area over a 30‐s sampling window. Dividing femoral arterial blood flow by mean arterial pressure provided a measure of limb vascular conductance. Femoral artery blood velocity and diameter were measured in duplicate during the baseline period and every minute during IPTL.

### 2.8 Cerebrovascular haemodynamics

Cerebral blood velocity of the right middle cerebral artery (MCA), as a proxy of global cerebral blood flow, was measured continuously using transcranial Doppler ultrasound (9F TCD, 2 MHz probe, Multigon USA, Flint, TX, USA). The transtemporal window was used for insonation in all patients, and confirmation of the MCA was determined using previously reported methods on direction, depth and velocity (Willie et al., 2011). Briefly, the probe was placed ∼1 cm anterosuperior to the tragus, above the zygomatic arch. When searching for the MCA, the internal carotid artery bifurcation to the MCA and anterior cerebral artery (ACA) were first identified. From here, insonation was translocated slightly superiorly, and the depth and angle were adjusted to yield signal towards the probe, differentiating from ACA. These parameters of insonation were confirmed to the ‘standardized’ expected depths (∼25–50 mm) and peak velocities (∼40–90 cm s^−1^) of the MCA in healthy adults aged 18–30 years (Alwatban et al., 2021; Willie et al., 2011). The cerebrovascular conductance index (CVCi) was calculated by dividing MCA blood velocity by mean arterial pressure. The pulsatility index (PI) was calculated as: (systolic MCA blood velocity minus diastolic MCA blood velocity)/mean MCA blood velocity (Skow et al., 2022).

### 2.9 Pressure threshold loading

The IPTL was performed on a custom‐built pressure threshold loading device based on the design by Eastwood and Hillman (1995). The inspired circuit of the device was sealed until enough negative pressure was generated to lift a weighted valve, allowing air to flow freely. Expiration was unobstructed throughout. The IPTL device was connected to a non‐rebreathing valve (Series 7200, Hans Rudolph, Shawnee, KS, USA) with ports at the mouth to sample *P*_MO_ and $P_{\mathrm{ET},CO_{2}}$. Participants were semi‐supine throughout a 5 min baseline period and 5 min of IPTL. During baseline, participants breathed freely on the mouthpiece connected to the IPTL device, with the valve held open for unimpeded airflow. During IPTL, breathing frequency was set at 15 breaths min^−1^, with a prolonged inspiratory duty cycle (inspired time/total breath time = 0.7), and paced via audio metronome. The valve was set to open at ∼60% MIP, and a secondary target of 60% maximal *P*_DI_ was set. Participants were given real‐time visual feedback on *P*_MO_ and *P*_DI_, showing both the target and continuous pressure traces. Additionally, participants were encouraged by the study team if a breath did not meet the targeted pressure. The $P_{\mathrm{ET},CO_{2}}$ was monitored throughout IPTL, In the event of hypocapnia, defined as a decrease in $P_{\mathrm{ET},CO_{2}}$ from baseline by ≥3 mmHg, trace amounts of additional CO_2_ were titrated manually into the inspired circuit.

In order to familiarize our participants with the protocol, participants engaged in two independent familiarization trials, with the first based on duty cycle and the second based on pressure generation. Duty cycle familiarization was performed on an open mouthpiece with the audible metronome playing. After several breaths, participants were shown their flow trace. This was performed until participants felt comfortable with the timing of breaths and the research team was satisfied that participants could consistently match inspiration with the metronome. To familiarize participants with the pressure thresholds of the IPTL task, the research team first explained the visual feedback channels of *P*_MO_ and *P*_DI_. Distinctly coloured bands were used to orient participants to the limits of pressure to be generated. Participants would inspire against the occluded IPTL device set to an estimate of target *P*_MO_ and were instructed to ‘feel when the valve opens and air is coming through’. This was repeated in small bouts (one to three breaths) to adjust the weights to be adequate for the desired pressure. After this initial familiarization, participants would practice a ‘diaphragmatic’ breathing pattern by placing one hand on the belly and the other on the ribs while engaging in tidal breathing off mouthpiece. Participants were told to feel ‘the hand on the belly moving and the hand on the ribs remaining somewhat still’ over the cycle of a breath. Participants were shown the *P*_DI_ trace during these tidal breaths and asked to note how the pressure increased over inspiration and the difference between their tidal breathing pressure and the desired *P*_DI_ target. After participants were comfortable with these three independent tasks, a final familiarization incorporating all three was performed. This familiarization task was essentially a practice of the IPTL task over short bouts (three to six breaths). Participants rested for a minimum of 1 min between each familiarization bout. When researchers were satisfied that the participant could consistently maintain the parameters of the task for three to six breaths and the participant felt comfortable proceeding, the experimental trials began. Overall, familiarization took between ∼10 and 45 min for participants.

### 2.10 Statistical analyses

Sample size calculation was performed using G*Power. An estimated effect size of 0.26, based on the increase in mean arterial pressure with 5 min of IPTL from Archiza et al. (2021), an α of 0.05, power (1 − β) of 0.80 across the two groups with all measurement points yields a total sample size of 18. No cerebrovascular responses to IPTL were available to estimate sample size; therefore, mean arterial pressure was used as an indicator that the respiratory muscle metaboreflex was activated. Sample size was increased to account for potential participant dropout and new outcome measures.

Data were tested for normality using a Shapiro–Wilk test. All reported data were normally distributed. A two‐way (time × sex) repeated‐measures ANOVA was used to compare cardiopulmonary outcomes. The main effect of time served our primary purpose to investigate the overall effect of IPTL on these cardiopulmonary outcomes, whereas the main effect of sex and the interaction fulfilled our exploratory analysis into sex‐based differences. This decision was made to minimize introduction of additional family‐wise error through multiple comparisons and statistical tests. Tukey's *post hoc* analyses were applied based on omnibus ANOVA results to investigate main effects. Descriptive characteristics between males and females were compared using Student's unpaired *t*‐test. A value < 0.05 was considered significant for all comparisons. Analyses were completed in R (v.4.4.1; R Foundation for Statistical Computing, Vienna, Austria), and data are presented as the mean ± SD.

## 3 RESULTS

### 3.1 Participant characteristics

A total of 28 participants [14 female (F) and 14 male (M)] were recruited to take part in this study. Of the 28, four did not complete the study for the following reasons: discomfort completing the IPTL task (1F:1M), exclusion based on spirometry (FEV_1_/FVC < 80% predicted; 1M), or discomfort with the oesophageal catheter (1F). This resulted in a total of 24 participants (12F:12M) included in the analysis. For cerebrovascular outcomes, an additional four participants (2M:2F) were excluded owing to incomplete transcranial Doppler ultrasound data.

When examining participant data between sexes, male participants were, on average, taller and heavier and had greater pulmonary function values in absolute terms. Differences in lung function were not present when evaluated as %predicted values. Baseline heart rate and mean arterial pressure were not different between groups. Participant characteristics are presented in Table 1.

**TABLE 1:** Participant characteristics at rest.

Characteristic	Females	Males	*p*‐value
*n*	12	12	–
Age, years	34 ± 10	31 ± 8	0.440
Height, cm	164 ± 7	182 ± 9	<0.0001
Weight, kg	63 ± 8	83 ± 10	<0.0001
BMI, kg m^−2^	23 ± 2	25 ± 3	0.0248
Pulmonary function			
FEV_1_, L (%predicted)	3.34 ± 0.40 (110 ± 11)	4.83 ± 0.52 (111 ± 16)	<0.0001 (0.786)
FVC, L (%predicted)	4.02 ± 0.56 (112 ± 15)	5.90 ± 0.83 (112 ± 14)	<0.0001 (0.943)
FEV_1_/FVC (%predicted)	0.84 ± 0.06 (99 ± 7)	0.83 ± 0.08 (100 ± 7)	0.718 (0.826)
MIP, cmH_2_O (%predicted)	98 ± 19 (113 ± 19)	138 ± 30 (111 ± 23)	<0.0001 (0.846)
*P*_DI,MAX_, cmH_2_O	80 ± 28	120 ± 37	0.0067
Resting cardiovascular function			
Heart rate, beats min^−1^	70 ± 11	71 ± 11	0.743
Systolic blood pressure, mmHg	116 ± 14	128 ± 16	0.061
Diastolic blood pressure, mmHg	77 ± 11	80 ± 8	0.552
Mean arterial pressure, mmHg	90 ± 12	96 ± 8	0.191

*Note*: Sex distribution data are presented as counts and all other data as the mean ± SD. Abbreviations: BMI, body mass index; FEV_1_, forced expired volume in 1 s; FVC, forced vital capacity; MIP, maximal inspiratory mouth pressure.

**FIGURE 1:**
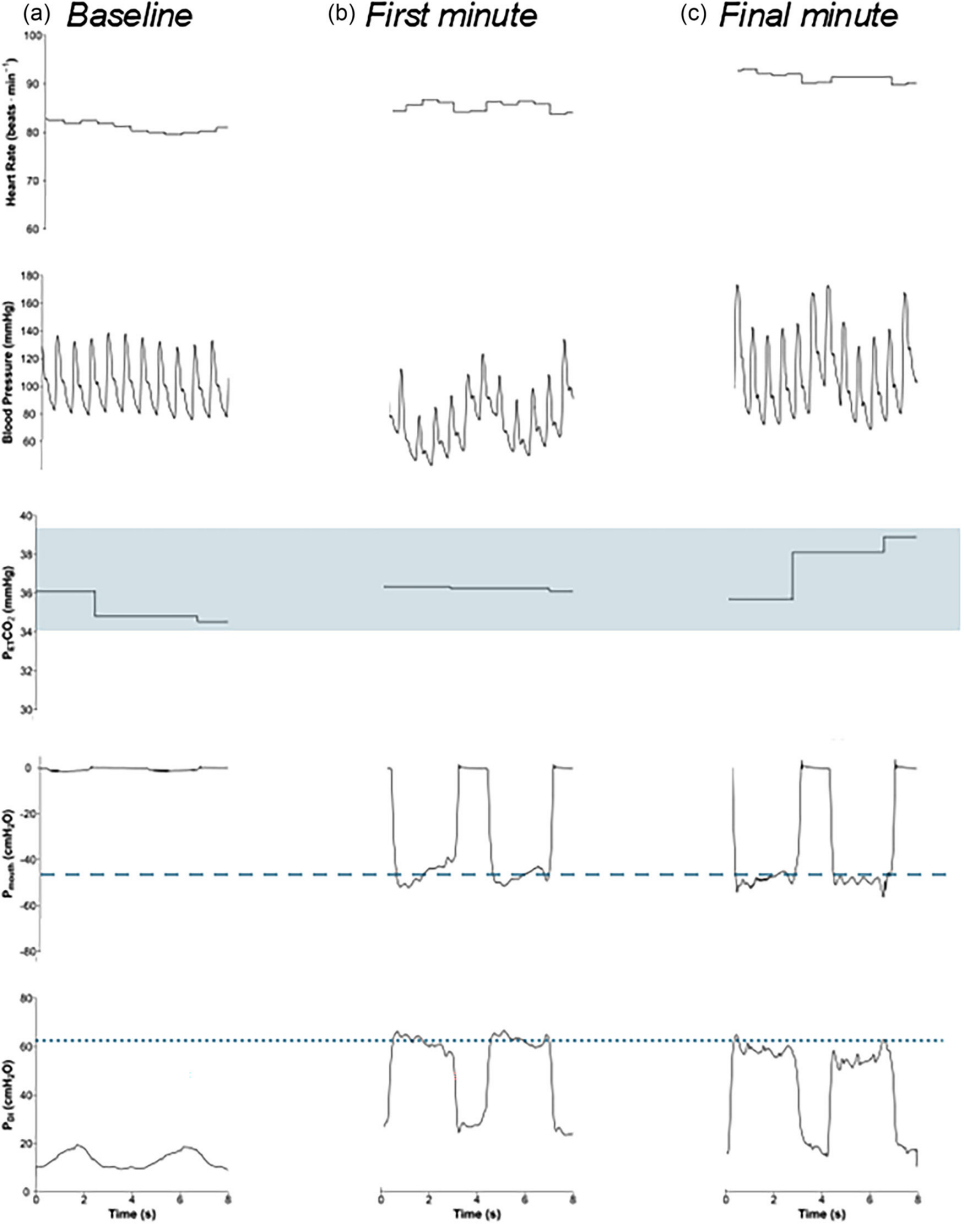
Representative traces of select variables during pressure threshold loading. Heart rate, blood pressure, end‐tidal CO_2_ partial pressure ($P_{\mathrm{ET},CO_{2}}$), mouth pressure (*P*_mouth_) and transdiaphragmatic pressure (*P*_DI_) at baseline (a) and during the first (b) and final (c) minute of inspiratory pressure threshold loading.

On average, participants targeted −71 ± 20 cmH_2_O mouth pressure (F, −60 ± 11 cmH_2_O vs. M, −83 ± 19 cmH_2_O; *p =* 0.005) and 60 ± 24 cmH_2_O transdiaphragmatic pressure (F, 48 ± 18 cmH_2_O vs. M, 73 ± 24 cmH_2_O; *p =* 0.0178) during their IPTL task. Respiratory variables during IPTL are presented in Table 2. All participants completed the full 5‐min IPTL trial. Representative traces of select cardiopulmonary variables during IPTL are shown in Figure 1. On average, males performed more inspiratory work than females (PTP_MO_, 11 129 ± 4115 vs. 7251 ± 2856 cmH_2_O s^−1^, respectively, *p =* 0.014) after 5 min of IPTL; however, when comparing PTP_DI_ between sexes there was no observed difference (6389 ± 2359 vs. 7879 ± 1607 cmH_2_O s^−1^, respectively, *p =* 0.0844)

**TABLE 2:** Respiratory variables during inspiratory pressure threshold loading.

		Time						*p‐*Value		
Variable	Sex	Baseline	1st min	2nd min	3rd min	4th min	5th min	Sex	Time	Interaction
*F*_b_, breaths min^−1^	F	14 ± 3	15 ± 1	16 ± 1	16 ± 1	16 ± 1	15 ± 2	<0.0001	0.032	0.010
	M	17 ± 5	15 ± 3	15 ± 2	16 ± 1	15 ± 2	15 ± 2			
*T*_I_/*T*_TOT_	F	0.61 ± 0.19	0.72 ± 0.10	0.70 ± 0.12	0.69 ± 0.11	0.70 ± 0.11	0.71 ± 0.11	0.790	0.0582	0.517
	M	0.60 ± 0.17	0.76 ± 0.07	0.75 ± 0.11	0.76 ± 0.15	0.78 ± 0.07	0.77 ± 0.06			
$P_{\mathrm{ET},CO_{2}}$, mmHg	F	36 ± 5	35 ± 4	35 ± 4	34 ± 6	36 ± 5	36 ± 5	0.753	0.491	0.160
	M	37 ± 3	39 ± 5	39 ± 4	39 ± 3	40 ± 3	40 ± 4			

*Note*: All data are presented as the mean ± SD. Abbreviations: F, female; *F*_b_, breathing frequency; M, male; $P_{\mathrm{ET},CO_{2}}$, partial pressure of end‐tidal carbon dioxide; *T*_I_, inspiratory time; *T*_TOT_, total breath time.

### 3.2 Cerebral haemodynamics

**FIGURE 2:**
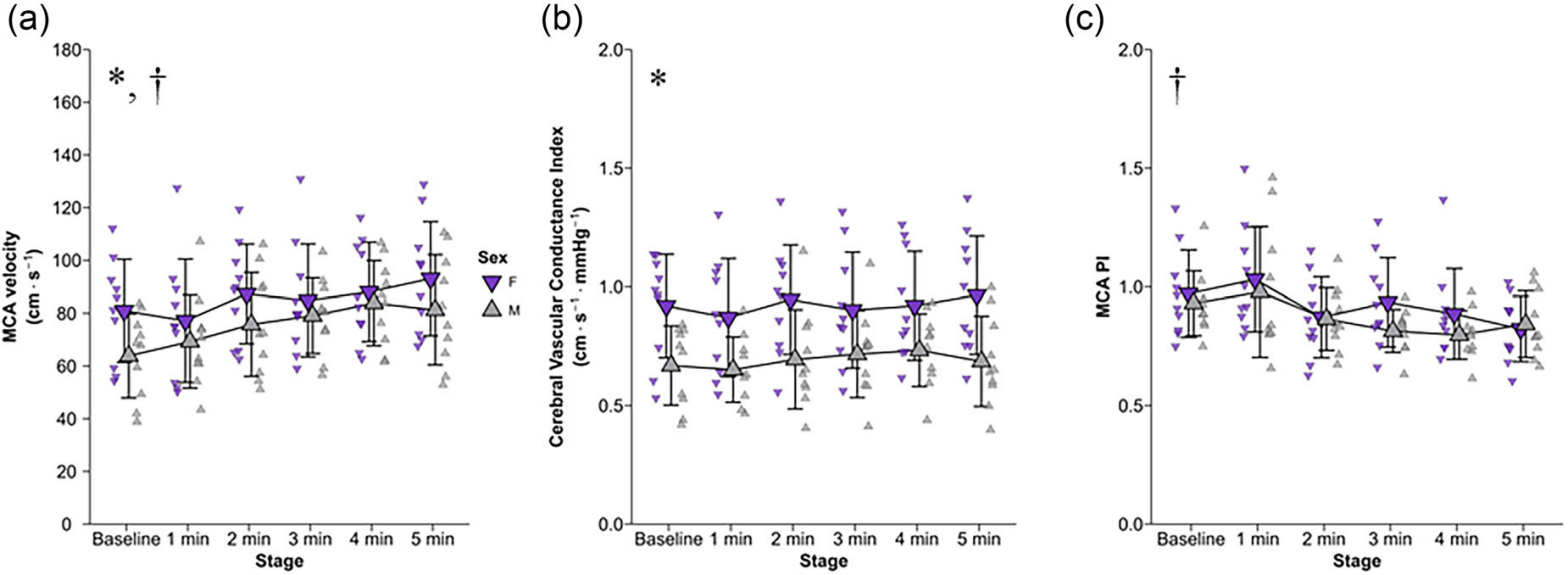
Cerebrovascular responses to inspiratory pressure threshold loading. Mean (large shapes) with SD and individual (small shapes) data for blood velocity (a), cerebrovascular conductance index (b) and pulsatility index (c) measured at the right middle cerebral artery in response to inspiratory work. *p < 0.05, main effect of sex; †*p <* 0.05, main effect of time.

There were no observed interaction effects of time × sex for right MCA velocity (*p =* 0.4252); however, right MCA velocity was greater in females versus males (*p =* 0.0466) and increased over time (*p =* 0.0153). When accounting for blood pressure, no interaction effects were observed (sex × time, *p =* 0.650; Figure 2a), but MCA CVCi was greater in females compared with males (main effect of sex, *p =* 0.0072). The CVCi was maintained during IPTL (main effect of time, *p =* 0.416). Furthermore, no significant interaction effects (*p =* 0.637) were observed in right MCA PI (Figure 2b), nor was PI different between sexes (*p =* 0.595), but a main effect of time was observed (*p =* 0.0131).

### 3.3 Peripheral haemodynamics

**FIGURE 3:**
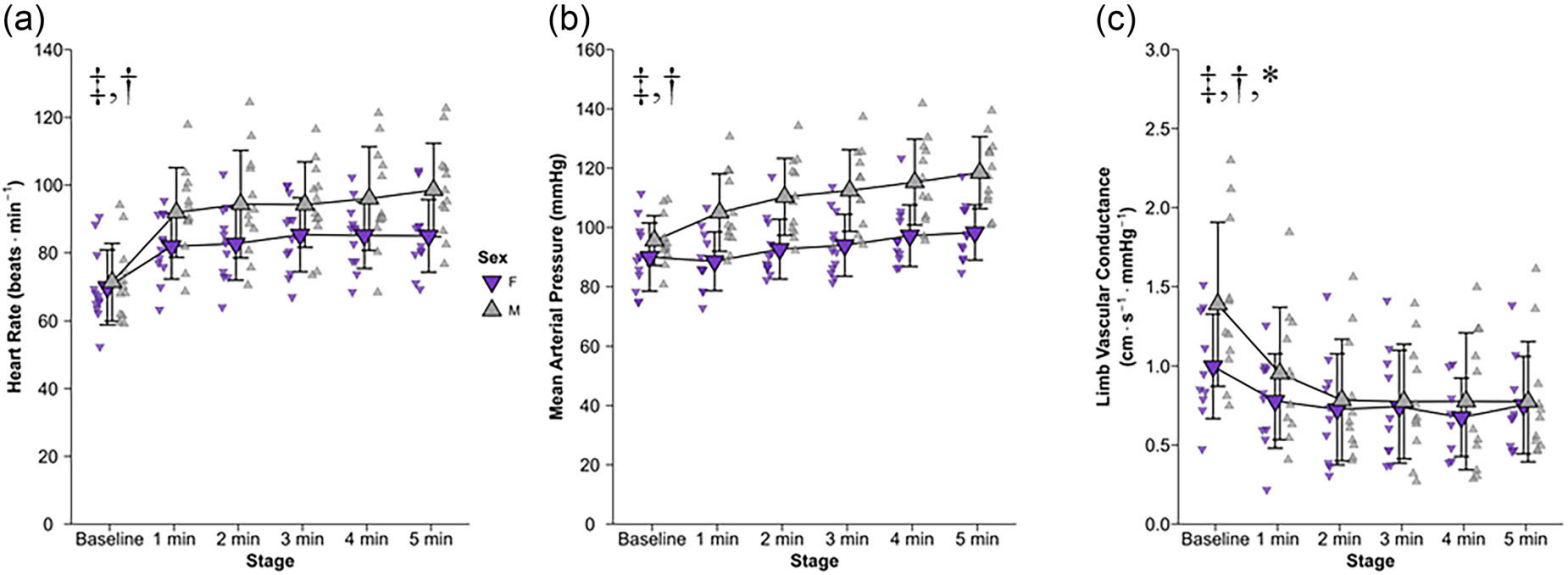
Peripheral haemodynamics during inspiratory pressure threshold loading. Mean (large shapes) with SD and individual (small shapes) data for heart rate (a), mean arterial pressure (b) and limb vascular conductance (c) in response to inspiratory work. *p < 0.05, main effect of sex; †*p <* 0.05, main effect of time; ‡*p <* 0.05, significant sex × time interaction effect.

Haemodynamic variables during IPTL are shown in Table 3. There was a significant interaction effect (*p <* 0.0001) indicating that the heart rate increase during IPTL was greater in males compared with females (Figure 3a). A main effect of time (*p <* 0.0001) but not sex (*p =* 0.761) was observed for heart rate. Likewise, a significant interaction (*p <* 0.0001) and main effect of time (*p <* 0.0001) were observed with respect to mean arterial pressure (Figure 3b), without an observed main effect of sex (*p =* 0.238).

**TABLE 3:** Haemodynamic variables during inspiratory pressure threshold loading.

		Time						*p‐*Value		
Variable	Sex	Baseline	1st min	2nd min	3rd min	4th min	5th min	Sex	Time	Interaction
Heart rate, beats min^−1^	F	70 ± 11	82 ± 10	83 ± 11	85 ± 11	85 ± 10	85 ± 11	0.761	<0.0001	<0.0001
	M	71 ± 11	92 ± 13	94 ± 16	94 ± 13	96 ± 15	99 ± 14			
MAP, mmHg	F	90 ± 12	89 ± 10	93 ± 10	94 ± 10	97 ± 10	98 ± 9	0.238	<0.0001	<0.0001
	M	96 ± 8	105 ± 13	110 ± 13	112 ± 14	115 ± 15	118 ± 12			
LVC, mL s^−1^ mmHg^−1^	F	1.00 ± 0.33	0.78 ± 0.30	0.73 ± 0.35	0.74 ± 0.36	0.68 ± 0.25	0.75 ± 0.31	0.0179	<0.0001	0.005
	M	1.39 ± 0.52	0.95 ± 0.42	0.78 ± 0.39	0.78 ± 0.36	0.78 ± 0.43	0.77 ± 0.38			
MCA CVCi, cm s^−1^ mmHg^−1^	F	0.93 ± 0.21	0.90 ± 0.26	0.96 ± 0.22	0.94 ± 0.26	0.92 ± 0.22	0.96 ± 0.24	0.0072	0.416	0.650
	M	0.67 ± 0.17	0.65 ± 0.14	0.69 ± 0.21	0.72 ± 0.18	0.73 ± 0.15	0.69 ± 0.19			
MCA PI	F	0.94 ± 0.20	0.99 ± 0.24	0.87 ± 0.16	0.90 ± 0.21	0.88 ± 0.18	0.83 ± 0.13	0.595	0.0131	0.637
	M	0.93 ± 0.14	0.98 ± 0.28	0.86 ± 0.13	0.81 ± 0.09	0.80 ± 0.10	0.84 ± 0.14			

*Note*: All data are presented as the mean ± SD. Abbreviations: CVCi, cerebrovascular conductance index; F, female; LVC, limb vascular conductance; M, male; MAP, mean arterial pressure; MCA, middle cerebral artery; PI, pulsatility index.

When examining leg vascular conductance at the femoral artery, we observed a significant interaction effect of sex × time (*p =* 0.005) along with main effects of sex (*p =* 0.0179) and time (*p <* 0.0001). These findings suggest that the average conductance over the IPTL protocol was greater in males and decreased to a greater extent compared with the change observed in females (Figure 3c).

## 4 DISCUSSION

### 4.1 Main findings

The primary aim of this study was to explore the cerebrovascular responses to inspiratory muscle work. Our main finding is that cerebrovascular haemodynamics (MCA CVCi and PI) are maintained in response to a respiratory muscle metaboreflex that elicits a decrease in conductance of the resting peripheral muscle. Furthermore, these data suggest that previously noted sex differences in the cardiovascular response to high inspiratory work (Benbaruj et al., 2024; Geary et al., 2019; Leahy et al., 2024; Smith, Broxterman et al., 2016) might not extend to cerebrovascular control.

### 4.2 Cerebrovascular responses to inspiratory muscle work

Inspiratory muscle work does not appear to have a significant effect on indices of cerebral haemodynamics. During whole‐body exercise, the distribution of cardiac output can play a role in cerebral blood flow (Smith, Wildfong et al., 2016). With the speculated redistribution of cardiac output observed as a result of the respiratory muscle metaboreflex (Sheel et al., 2018), it is plausible to think that cerebral blood flow could be impacted in a similar manner. Conversely, given that cerebral blood flow is tightly regulated to maintain critical cognitive function, it stands to reason that these haemodynamic changes observed in the periphery would not be mirrored in the brain. Our findings favour the latter hypothesis; given significant changes in heart rate, blood pressure and resting limb conductance, no changes in CVCi were observed. In response to IPTL, mean MCA velocity increased, possibly owing to increases in total cardiac output. Overall, MCA CVCi was not affected by IPTL, suggesting that the increases in MCA velocity were proportional to the changes in mean arterial pressure. Investigating the relationship between mean arterial pressure and cerebral blood flow through transfer function analysis is a plausible direction for future studies to investigate cerebral autoregulation further. The MCA PI, serving as an index of cerebral arteriole resistance, decreased as IPTL progressed. This was an unexpected finding, because previous literature in resistance exercise has demonstrated an increase in MCA PI during an acute bout of resistance exercise (Marôco et al., 2023). The discrepancies between our results might be attributable to the interventions used. A combination of maximal intensity exercise using a larger muscle mass, as done by Marôco et al. (2023), might have more pronounced effects than our submaximal respiratory muscle‐focused intervention. Further work is needed to gain a better understanding of how the distribution of blood flow is regulated among large exercising muscles, respiratory muscles and the cerebral vasculature.

With respect to sex differences, we observed an overall greater MCA velocity and CVCi in our female participants compared with their male counterparts. The lack of significant interaction effects suggests that biological sex does not exacerbate the cerebrovascular response to high‐intensity inspiratory work. Our findings are consistent with previous research in that, at rest, cerebral blood velocity and PI tend to be greater in females compared with males (Alwatban et al., 2021). Furthermore, MCA velocity increased to a similar degree in both sexes after 5 min of IPTL, which can be compared with previous findings from both handgrip (Joshi & Edgell, 2019) and whole‐body exercise (Chamoun et al., 2023). The source of this sex difference is not well understood, in part, owing to a lack of available literature in which sex differences are a primary outcome. Given that we have observed a sex difference in the systemic (i.e. peripheral and central) but not cerebral haemodynamics, it is plausible to infer that there are key differences in cerebral blood flow regulation between sexes. Sex differences in cerebral haemodynamics might be related to morphological differences in the cerebral vasculature. On average, the diameter of the middle cerebral artery is larger in males compared with females (Müller et al., 1991). It is also possible that circulating sex hormones, primarily oestrogens through β‐adrenergically mediated vasodilatory effects, can influence cerebral blood flow, as seen in systemic haemodynamics (Hart et al., 2011). Our speculation highlights the need for additional, mechanistic studies to gain a better understanding of the role of biological sex in regulating cerebral blood flow, with a focus on the integrative response to exercise.

### 4.3 Central and peripheral haemodynamic responses to inspiratory work

The impact of inspiratory muscle work on haemodynamics, including blood pressure, heart rate and blood flow, has routinely demonstrated an integrative response between respiratory and cardiovascular systems. It is thought that the function of this respiratory muscle metaboreflex is to distribute cardiac output adequately to demanding muscles and might preferentially direct cardiac output to the respiratory muscles at the expense of the exercising limbs (Sheel et al., 2018). Although these metaboreflex effects have been observed during whole‐body exercise (Harms et al., 1997), the performance of isolated inspiratory muscle work, such as through IPTL, allows for an investigation of the respiratory muscle metaboreflex in the absence of a larger exercise pressor response. Our findings mirror those of previous studies (Benbaruj et al., 2024; Leahy et al., 2024, 2023; Sheel et al., 2001; Smith, Broxterman et al., 2016; St Croix et al., 2000) in that fatiguing inspiratory work evokes cardiovascular effects such as an increase in heart rate and mean arterial pressure along with a decrease in blood flow of the inactive leg. These cardiovascular effects are likely to be facilitated through the activation of group III and IV afferent fibres (Haouzi et al., 1999). Although respiratory muscle fatigue was not measured directly in this study, we believe that our IPTL protocol could be considered a ‘fatiguing’ protocol. First, the tension–time index of the diaphragm, the product of the inspired duty cycle (0.7) and relative transdiaphragmatic pressure (60%maximal *P*_DI_), used in this study was 0.42 and would exceed the threshold diaphragm tension–time index of 0.2 that has been observed to impede diaphragm blood flow and create a fatiguing muscle environment (Bellemare et al., 1983). Second, previously, a 5‐min IPTL protocol in similar conditions has been used successfully to fatigue the diaphragm and elicit inspiratory muscle metaboreflex responses (Archiza et al., 2021), although it should be noted that while the relative intensities were similar, the absolute targeted *P*_DI_ and the corresponding cumulative PTP_DI_ at end‐task were lower in our study.

There are well‐reported sex differences in respiratory system morphology that lead to functional differences in respiratory system function, which are particularly evident during exercise (Dominelli & Molgat‐Seon, 2022). Germane to the present study, females appear to have an attenuated respiratory muscle metaboreflex response compared with males, strongly supported by an attenuated sympathetic response, when performing similar relative and absolute inspiratory work (Leahy et al., 2024). A blunted respiratory muscle metaboreflex response in females is also evident through lesser increases in heart rate and mean arterial pressure compared with their male counterparts. This has been observed at similar relative (Smith, Broxterman et al., 2016; Welch et al., 2018) and absolute (Geary et al., 2019) intensities. The results of our study corroborate these findings, supporting the evidence to say that young females have an attenuated cardiovascular response to fatiguing inspiratory work.

Blood flow to the resting limb has also been found to decrease in response to inspiratory work (Sheel et al., 2001). Furthermore, previous studies have found that the relative decrease in blood flow at the femoral artery is greater in males compared with females, further supporting the notion of an attenuated respiratory muscle metaboreflex in females (Smith, Broxterman et al., 2016). We too observed this sex difference in changes to resting limb conductance. At rest, conductance was greater in males compared with females, and when performing IPTL the conductance fell in both groups; however, the decrease in conductance was greater in males than in females. Considering that vascular conductance is composed of two components, mean arterial pressure and limb blood flow, we can examine these components to describe our findings better. We posit that the reduction in conductance was attributable to a combination of an increase in mean arterial pressure and a decrease in blood flow, and the attenuated increase in mean arterial pressure observed in females might contribute to improved conductance relative to males.

Although beyond the scope of the present study, the precise mechanisms contributing to these sex differences merit discussion. The prevailing framework of the respiratory muscle metaboreflex begins with the stimulation of afferent fibres originating in the diaphragm. These group III and IV afferents are known to be stimulated with exercise (Adreani et al., 1997) and, specifically in the diaphragm, have also been shown to be metabolically sensitive. The metabolites of consequence for activating the metaboreflex are thought to be associated with skeletal muscle fatigue (Rodman et al., 2003). There is evidence that females are more resistant to respiratory muscle, specifically diaphragm, fatigue as a result of both whole‐body exercise (Guenette et al., 2010; Ramsook et al., 2022) and isolated inspiratory work (Ramsook et al., 2023; Welch et al., 2018) at relative intensities. This sex difference in respiratory muscle fatigue might be the result of an improved ability to clear fatigue‐associated metabolites through mechanisms such as greater diaphragm perfusion (Bird et al., 2025; Lublin et al., 1995), thereby providing a lesser stimulus to the afferents triggering the metaboreflex. Further work is required to elucidate these mechanisms and their potential contributions to the metaboreflex.

### 4.4 Methodological considerations

Our IPTL protocol was fixed at 5 min. Fixed, 5‐min tasks have previously elicited the respiratory muscle metaboreflex and been used to assess sex‐based differences (Geary et al., 2019; Smith, Broxterman et al., 2016). Nonetheless, it is possible that allowing participants to continue to perform IPTL until exhaustion might influence the respiratory muscle metaboreflex responses observed by allowing a greater accumulation of metabolites associated with skeletal muscle fatigue.

The IPTL task presented in this study used a relative intensity to elicit the respiratory muscle metaboreflex response, and there is an argument to be made that the absolute intensity of a task must also be acknowledged when examining sex differences in metaboreflex responses (Lee & Millar, 2024). Our decision to use a relative intensity was made to facilitate comparisons with previous research by using a similar study design (Leahy et al., 2023; Welch et al., 2018). Concerning inspiratory work, the apparent blunted cardiovascular response in females is present both at relative (60% maximal inspiratory pressure) and absolute load (Benbaruj et al., 2024).

Cerebrovascular control can be influenced by several factors, the most potent of which appears to be arterial CO_2_ (Hoiland et al., 2019). In the present study, we attempted to avoid hypocapnia by manually introducing CO_2_ into the inspired circuit; however, we were unable to remove CO_2_ to make the task completely isocapnic. Although there was no statistically significant change in $P_{\mathrm{ET},CO_{2}}$ during IPTL in either males or females, there is a possibility that minor increases in $P_{\mathrm{ET},CO_{2}}$ were physiologically relevant to influence cerebral blood velocity. Dynamically adjusting the composition of the inspired gas to clamp $P_{\mathrm{ET},CO_{2}}$ effectively could alleviate this concern entirely; however, these systems are not without limitations of their own (Tymko et al., 2015).

It is also worth reiterating that the cerebrovascular conclusions are assuming a stable vessel diameter. Gold‐standard MRI studies have demonstrated that MCA vessel diameter remains stable within a range of CO_2_ fluctuations (approximately ±10 mmHg different from baseline $P_{\mathrm{ET},CO_{2}}$; Ainslie & Hoiland, 2014). Within the present study, the average $P_{\mathrm{ET},CO_{2}}$ remained within ∼3 mmHg from baseline values, yielding less concern for changes in MCA vessel diameter. In terms of blood pressure sensitivity, we are unsure of data suggesting MCA vessel diameter changes in response to normal fluctuations in blood pressure. Probably, in extreme hypo‐ and/or hypertensive states we expect MCA vessel diameters to be affected, but given the widespread validity of use in MCA insonation during activities such as cycling exercise, we would not suspect blood pressure to influence diameter to a significant degree within the relatively small range that this study invoked.

Lastly, our study was designed with the primary purpose of investigating the overall influence of the respiratory muscle metaboreflex on cerebral haemodynamics, with an exploratory aim to investigate sex differences. Our findings regarding sex differences are therefore to be interpreted with the appropriate caution and can be used as groundwork for future studies.

## 5 CONCLUSION

Our findings suggest that the respiratory muscle metaboreflex, elicited through high inspiratory work, does not result in a decrease in cerebrovascular conductance index, contrasting the observed decrease in conductance of the resting limb. We believe that this reflects the idea that cerebrovascular conductance is prioritized to maintain homeostasis and is therefore less likely to be impacted negatively by systemic alterations to cardiovascular function. Furthermore, although females had a higher cerebrovascular conductance index, sex‐based differences in the cardiovascular response to the respiratory muscle metaboreflex do not appear to influence cerebrovascular haemodynamics, although these sex‐based inferences would benefit from future studies with a larger sample size.

## AUTHOR CONTRIBUTIONS

Andrew H. Ramsook, Chad C. Wiggins and Michael J. Joyner conceptualized and designed the work. Andrew H. Ramsook, Morgan A. Hughes, Wyatt W. Pruter, Kevin L. Webb, Gabrielle A. Dillon, Ali Ataie and Sarah E. Baker collected the data. Andrew H. Ramsook, Morgan A. Hughes and Wyatt W. Pruter analysed the data. All authors contributed to the interpretation of data. All authors critically revised the manuscript for content, approved the final version for submission and publication and agree to be accountable for all aspects of the work in ensuring that questions related to the accuracy or integrity of any part of the work are appropriately investigated and resolved. All persons designated as authors qualify for authorship, and all those who qualify for authorship are listed.

## CONFLICT OF INTEREST

None declared.

## Data Availability

Data sharing must be compliant with all applicable Mayo Clinic policies. Individual data may be made available to approved investigators for secondary analyses upon reasonable request and approval from Mayo Clinic Institutional Review Board.
